# Aquatic vegetation in response to increased eutrophication and degraded light climate in Eastern Lake Taihu: Implications for lake ecological restoration

**DOI:** 10.1038/srep23867

**Published:** 2016-04-04

**Authors:** Yunlin Zhang, Xiaohan Liu, Boqiang Qin, Kun Shi, Jianming Deng, Yongqiang Zhou

**Affiliations:** 1 Taihu Laboratory for Lake Ecosystem Research, State Key Laboratory of Lake Science and Environment, Nanjing Institute of Geography and Limnology, Chinese Academy of Sciences, Nanjing 210008, P. R. China; 2 University of Chinese Academy of Sciences, Beijing 100049, P. R. China

## Abstract

Terrestrial and aquatic ecosystem degradation is widely recognized as a major global environmental and development problem. Although great efforts have been made to prevent aquatic ecosystem degradation, the degree, extent and impacts of this phenomenon remain controversial and unclear, such as its driving mechanisms. Here, we present results from a 17-year field investigation (1998–2014) of water quality and a 12-year remote sensing mapping (2003–2014) of the aquatic vegetation presence frequency (VPF) in Eastern Lake Taihu, a macrophyte-dominated bay of Lake Taihu in China. In the past 17 years, nutrient concentrations and water level (WL) have significantly increased, but the Secchi disk depth (SDD) has significantly decreased. These changes were associated with increased lake eutrophication and a degraded underwater light climate that further inhibited the growth of aquatic vegetation. In Eastern Lake Taihu, increased nutrients, chlorophyll *a* and WL, and a decreased SDD were all significantly correlated with a decreased VPF. NH_4_^+^-N concentration and SDD/WL were the most important controlling factors for VPF. Therefore, increased anthropogenic nutrient inputs and a degraded underwater light climate surely result in a decreased VPF. These results elucidate the driving mechanism of aquatic vegetation degradation and will facilitate Lake Taihu ecological restoration.

Aquatic vegetation supports critical ecological services by providing the habitat for a diverse and economically important faunal community, sequestering carbon and nutrients, stabilizing sediment and shorelines^1^,^2^,^3^. In addition, aquatic vegetation is a biological indicator or sentinel of water quality and ecological value in aquatic ecosystems^1^,^4^. In particular, the presence of submerged aquatic vegetation (SAV) is associated with increased clarity and good water quality^1^,^2^. In lakes with a high biomass of SAV over large areas, the levels of nutrients and the phytoplankton concentrations in the water column are lower; therefore, the Secchi disk depth (SDD) is greater because SAV beds stabilize to prevent sediment resuspension, nutrient release and phytoplankton growth^5^,^6^. In contrast, if SAV is scarce or lost entirely, the same lakes may have high concentrations of nutrients, phytoplankton blooms, high turbidity and poor water quality^6^. Accordingly, a stable state shift theory has been developed to describe the shift between macrophyte-dominated clear water states and phytoplankton-dominated turbid water states in shallow lakes^7^,^8^.

Unfortunately, the aquatic vegetation of lake, coastal and marine ecosystems has undergone substantial degradation with the onrushing advance of human settlement and water resources exploitation throughout the world in past decades^1^,^9^,^10^,^11^,^12^. For example, a study over the past 100 years in shallow lakes showed that the majority of lakes have lost all or most of their macrophyte taxa and that species richness declined markedly in the 13 studied streams in Denmark based on a comparison of SAV in lakes and streams in 1896 and 1996^13^. In addition, *in situ* and hyperspectral multispectral infrared and visible imaging spectrometer (MIVIS) images acquired over a 13-year period in Lake Garda (Italy) indicated that nearly 98% of macrophyte meadows were lost and subsequently replaced by moderate to extremely sparse communities with densities from 10% to 40% and that well-established submerged macrophytes (including Charophytes) were replaced by opportunistic aquatic plant species^11^.

These changes have largely been attributed to eutrophication caused by increasing nutrients and sediments from the alteration of the surrounding catchments and their subsequent effects on water quality, *e.g*., reduced SDD and increased phytoplankton, hypoxia, and anoxia in surficial sediments^7^,^10^,^14^. In addition, habitat alteration due to reclamation, dredging and filling, aquaculture, and alien species invasion has also contributed to the decline of native aquatic vegetation species^15^,^16^,^17^,^18^.

In China, many shallow lakes have also undergone rapid aquatic vegetation degradation and water quality deterioration. The degradation process of aquatic vegetation is highly complex, and several affecting factors are often interlinked, making it important to consider both natural variation and anthropogenic effects. Many previous studies have tracked the spatio-temporal variability of the distribution of aquatic vegetation in shallow lakes and have explored the potential effects of environmental factors on aquatic vegetation using field investigations, experimental cultures, remote sensing and ecological modeling^19^,^20^,^21^. However, to our knowledge, no study combining long-term water quality observation data and aquatic vegetation mapping has been conducted to determine the mechanisms by which increased eutrophication and a degraded underwater light climate alter the trophic status of lakes and thus the aquatic vegetation. Thus, the understanding of aquatic vegetation dynamics and their determinants is critical to the management of shallow lakes because the flush growth and degradation of aquatic vegetation may control the ecosystem type, dynamics and lake water quality^4^.

Aside from conventional techniques, such as field mapping and photography, remote sensing can be an effective tool for mapping aquatic vegetation distributions over large scales. Many remote sensing techniques and methods, including spectral inversion, classification trees, the normalized difference vegetation index (NDVI), the floating algae index (FAI) and vegetation presence frequency (VPF), have been developed to accurately identify aquatic vegetation using different types of remote sensing imagery (TM, MODIS, MERIS and airborne data)^22^,^23^,^24^,^25^,^26^,^27^,^28^. In addition, long-term site-specific observations of water quality can generate long, data-rich time series and yield insights into the dynamics of water quality^6^,^29^.

Therefore, the purpose of this study was to combine long-term water quality monitoring data and remote sensing mapping of aquatic vegetation to elucidate the mechanisms by which increased eutrophication and a degraded underwater light climate had impacted environmental quality and thus degraded the aquatic vegetation in Eastern Lake Taihu, a shallow macrophyte-dominated bay located in the southeastern portion of Lake Taihu. We first explored the long-term dynamics of the habitat environment of the aquatic vegetation. We then used multi-year MODIS data to systematically quantify the interannual characteristics of the situation and strength of aquatic vegetation and the associated variability. Finally, we attempted to identify quantitative links between the responses of aquatic vegetation to increased eutrophication and the deteriorated underwater light climate. However, calibration, validation and quantitative comparisons of *in situ* aquatic vegetation to satellite remote sensing mapping had not been discussed in this study because they were beyond the scope of this manuscript; furthermore, these topics had been addressed in our previous study^26^.

## Materials and Methods

### Study region

**Figure 1:**
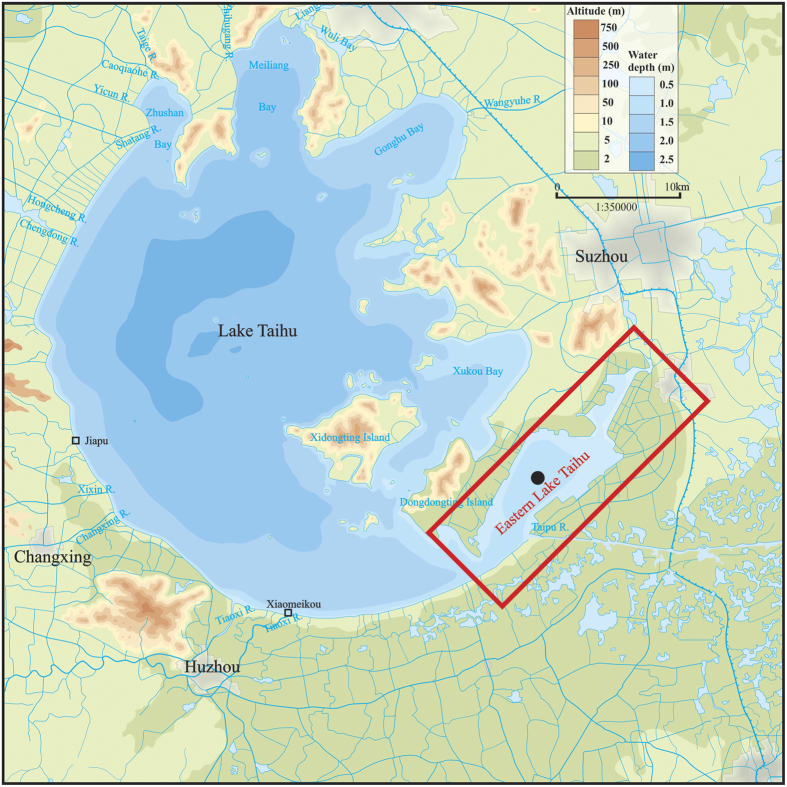
Schematic of Lake Taihu, the location of Eastern Lake Taihu (red block), the locations of the long-term site-specific observation site (•), and water level observation sites (□). This map was created using CorelDraw Graphic Suite X6 software (Corel Corporation, Ottawa, Canada, http://www.corel.com).

Lake Taihu, the third largest freshwater lake in China, is located in the Yangtze River Delta and has a water surface area of 2,338 km^2^, a mean depth of 1.9 m and a catchment area of 36,900 km^2^ 15. The lake basin includes the most developed region in China. Lake Taihu supplies drinking water to more than 10 million people within its watershed and is therefore of utmost importance for the surrounding residents and economic development. In the past few decades, Lake Taihu has experienced marked ecosystem degradation due to eutrophication, exhibiting frequent algal blooms every year from spring to autumn in the northern parts of the lake (Zhushan Bay, Meiliang Bay and Gonghu Bay) and a marked degradation of the aquatic vegetation in the southeastern parts of the lake (Xukou Bay and Eastern Lake Taihu) (Fig. 1)^15^,^30^,^31^.

Eastern Lake Taihu (30°58′-31°07′N, 120°25′-120°35′E) is a shallow macrophyte-dominated bay located in the southeastern portion of Lake Taihu (Fig. 1). According to field investigations and TM satellite image data in 1997, 95% of Eastern Lake Taihu is covered by aquatic vegetation^15^. Eastern Lake Taihu is connected to the main body of Lake Taihu by a narrow channel. Eastern Lake Taihu has a total length of 27.5 km, a maximum width of 9.1 km, a water area of 131 km^2^ and a mean water depth of 1.2 m.

### Sampling schedule

A 17-year (1998–2014) program of long-term site-specific observations has been conducted in Eastern Lake Taihu (Fig. 1, •). One water sample was collected during each season: a winter collection in February, a spring collection in May, a summer collection in August and an autumn collection in November. Surface water samples were collected in 2-L acid-washed bottles at a depth of 0.5 m and stored on ice while in the field. A standard 30-cm-diameter Secchi disk was used to measure SDD. All samples were transported to the Taihu Laboratory for Lake Ecosystem Research (TLLER) of the Chinese Academy of Sciences on the shores of Meiliang Bay and then immediately filtered or measured. All measurements were completed within 3–5 days.

### Measurement of water environment

The habitat environment of aquatic vegetation includes many aspects, such as water level (WL) (a substitute of water depth), nutrients, light availability, sediment, and predation pressure. Here, our study used observational data from TLLER to define critical SAV habitat requirements in terms of nine aquatic environmental and water quality variables: WL, SDD, total suspended matter (TSM), total nitrogen (TN), dissolved total nitrogen (DTN), ammonia (NH_4_^+^-N), total phosphorus (TP), dissolved total phosphorus (DTP), and chlorophyll *a* (Chl*a*). Samples for the determination of TSM, TDN, TDP, and Chl*a* concentrations were filtered using Whatman GF/F filters. Chl*a* was extracted with ethanol (90%) at 80 °C and analyzed spectrophotometrically at 665 nm and 750 nm using a Shimadzu spectrophotometer with a correction for phaeopigments (P*a*)^32^. Samples were filtered through pre-combusted (450 °C for 4 h) and pre-weighed Whatman GF/F filters to collect TSM of nominal sizes greater than 0.7 *μ*m. Samples were then dried (105 °C for 4 h) and weighed to calculate the TSM concentration.

Samples were analyzed for the concentrations of five nutrients—TN, TDN, NH_4_^+^-N, TP and TDP—according to the procedures for “Standard Methods for the Examination of Water and Wastewater”^33^. In detail, TN and DTN concentrations were measured by spectrophotometry at 210 nm after digestion. TP and TDP concentrations were determined by spectrophotometry at a wavelength of 700 nm, following the molybdenum blue method, after digestion with alkaline potassium persulfate (K_2_S_2_O_8_+NaOH). NH_4_^+^-N concentration was measured using a flow injection analyzer (Skalar SAN++, The Netherlands).

The daily WL of Lake Taihu was recorded at Jiapu and Xiaomeikou (Fig. 1, □). The yearly mean WL of Lake Taihu was calculated from the daily WL data for the two sites.

### Mapping presence frequency of aquatic vegetation

We used the FAI method^34^ to obtain the aquatic vegetation distribution for each image using MODIS imagery with a 250-m spatial resolution. A total of 1,125 high-quality MODIS images from 2003 to 2014 were selected after visual examination to exclude those substantially affected by clouds, sun glare, or thick aerosols. The FAI method has been found to be robust under virtually all conditions in the study area, including conditions affected by chromophoric dissolved organic matter, thick aerosols and frequent sun glare during the summer^31^. To discriminate the vegetation signal from the water matrix, we used the FAI as an indicator of aquatic vegetation. The area with aquatic vegetation can be distinguished from open water based on a critical FAI threshold. The FAI threshold of aquatic vegetation in Lake Taihu was set to −0.025, which has been determined and validated with the concurrent field data in our previous study^26^. Pixels with FAI values greater than −0.025 were defined as vegetation signals.

When the FAI of pixel *j* exceeded the FAI threshold of −0.025, the value of this pixel was set to 1 in the FAI layer; otherwise, the value of the pixel was set to 0. The VPF in each pixel was then evaluated in a given set of *n* images:


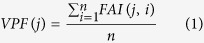


where VPF is the vegetation presence frequency of the pixel *j* in the image set of *n* and represents the proportion of pixel *j* with FAI = 1 in the total number of images (*n*). A detailed description of VPF is provided in our previous study^26^.

### Statistical analyses

Statistical analyses, including calculations of the average, maximum, and minimum values and linear and non-linear regressions, were performed using Statistical Program for Social Sciences (SPSS) 17.0 software. We performed correlation analyses using SPSS software to investigate the relationships between the variables. The differences in the parameters were assessed by one-way analysis of variance (ANOVA) (*p* < 0.05). The significance levels were reported as significant (*p* < 0.05) or non-significant (*p* > 0.05).

As the trends of environmental factors versus year in our study were hypothesized to be non-linear and the generalized additive model (GAM) provided a flexible and effective technique for modeling nonlinear time-series dataset^35^,^36^, GAMs were developed for WL, SDD, TSM, TN, DTN, NH_4_^+^-N, TP, DTP, and Chl*a*. Nonlinear fitting was performed using the GAM procedures with the “mgcv” package^37^ in R version 3.0.0^38^.

To determine the relative importance of environmental variables on the VPF, we used the concept of dispersion importance, *i.e*., we assessed the amount of variation explained by each predictor based on the multiple linear regression. TSM, TN, DTN, NH_4_^+^-N, TP, DTP, and Chl*a* were used as the predictors of relative importance. In addition, aquatic vegetation was largely determined by the light availability of the lake bottom^39^. Therefore, the ratio of SDD to WL (SDD/WL), representing the light availability of the lake bottom and considered a better predictor than the SDD or WL individually, was chosen as the predictor of the relative importance. First, we excluded the two auto-correlated variables (DTN and Chl*a*) according to the variance inflation factors (VIFs), and 10 was used as a threshold value^40^. Then, we constructed multiple linear regression model relating the VPF to the reserved environmental variables (namely, SDD/WL, TSM, TN, NH_4_^+^-N, TP, DTP) and then used the metric “lmg” with relaimpo package in R version 3.0.0^38^ to decompose the overall model *R*^2^ into nonnegative contributions for each predictor term^41^. The relative contribution of each predictor was then normalized and summed to the total *R*^2^ 41. This approach is based on sequential *R*^2^ but removes the dependence on orderings that bias stepwise regression by averaging over orderings, using simple unweighted averages^41^. This approach is recommended over other methods of decomposing the *R*^2^ among predictors because it uses both indirect and direct effects and adjusts for other predictors in the overall model^41^,^42^.

In addition, the relationship between VPF and habitat environment factors could be strongly non-linear and involved complex interactions that could not completely be explained by previously used linear statistical modeling approaches. The classification and regression tree (CART) model, as an efficient tool for extracting key variables and thresholds within a multivariate dataset, has been widely used in a variety of fields, such as environment and ecology^43^,^44^. The main aim of the CART model is to examine the complex relationships between habitat environment factors and VPF for investigating the relative importance of various factors. CART analysis was conducted using SPSS 17.0 software. Similar to the analysis of the relative importance, TSM, TN, DTN, NH_4_^+^-N, TP, DTP, Chl*a*, and SDD/WL were used as the predictors of the CART model.

## Results

### Long-term trends in the habitat environment of aquatic vegetation

**Figure 2:**
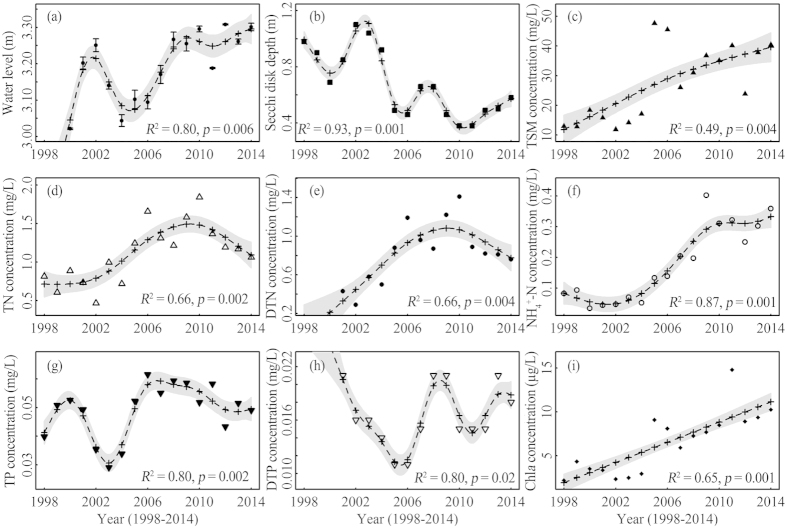
Long-term trends in the habitat environment of the aquatic vegetation for the nine variables: WL (**a**), SDD (**b**), TSM (**c**), TN (**d**), DTN (**e**), NH_4_^+^-N (**f**), TP (**g**), DTP (**h**), and Chl*a* (**i**). The dash lines indicate fitted values to the data for each given parameter versus year by GAMs.

The long-term site-specific observations showed that the nine aquatic environmental and water quality variables exhibited large fluctuations and different variation trends (Table 1, Fig. 2). Of the nine variables, WL, TSM, TN, NH_4_^+^-N, and Chl*a* exhibited a nearly linear increasing trend (Fig. 2a,c,f,i) based on the GAM time series analysis. In contrast, the GAM time series analysis indicated a nearly linear decreasing trend for SDD (Fig. 2b). However, there appeared to be a threshold for TN, DTN, TP, and DTP (Fig. 2d,e,g,h); our analysis suggested that the best models had a threshold time in 2009 or 2010, below which there was a marked increasing trend, and above which there was a slight decreasing trend. In summary, the nine variables formed two separate categories. Increased WL, TSM and Chl*a* concentrations and decreased SDD indicated the presence of a degraded underwater light environment in Eastern Lake Taihu. The increased TN, DTN, NH_4_^+^-N, TP, DTP, and Chl*a* concentrations indicated the intensified eutrophication in Eastern Lake Taihu in the past twenty years.

### Long-term trends and spatial distribution of aquatic vegetation

**Figure 3:**
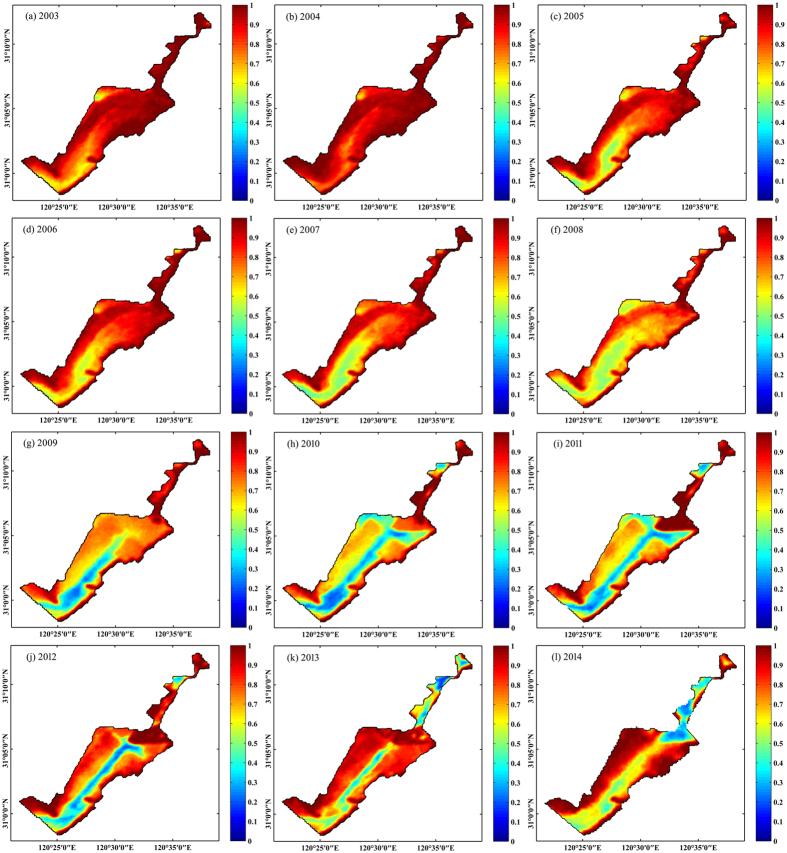
MODIS-Aqua-derived yearly climatology VPF from 2003 to 2014. 2003 (**a**), 2004 (**b**), 2005 (**c**), 2006 (**d**), 2007 (**e**), 2008 (**f**), 2009 (**g**), 2010 (**h**), 2011 (**i**), 2012 (**j**), 2013 (**k**), and 2014 (**l**).

**Figure 4:**
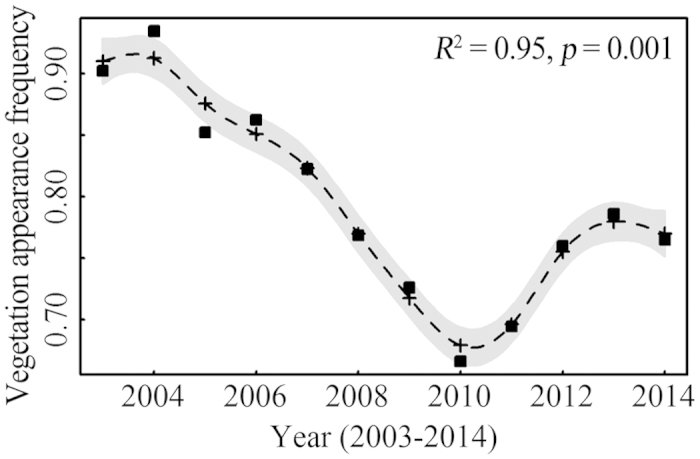
Long-term trends in the VPF from 2003 to 2014 in Eastern Lake Taihu. The dash line indicates fitted values of VPF data versus year by GAM.

More than 10 years of VPF calculated from a total of 1,125 high-quality MODIS images allowed us to recognize the temporal, spatial dynamics and long-term variation trend. The aquatic vegetation of Eastern Lake Taihu changed considerably over the study period, with the VPF declining significantly and nearly linearly from 2003 to 2014, as shown by the GAM time series analysis (Figs 3 and 4). The maximal VPF of 0.935 in 2004, based on remote sensing mapping, was lower than the value of 0.95 reported in 1997^15^. However, the GAM time series analysis indicated that there was no consistent decreasing trend from 2003 to 2014. From 2003 to 2010, the VPF rapidly decreased from 0.902 to 0.666 (26.2% annually) (Fig. 4). Then, the VPF slowly increased to 0.786 in 2013 and decreased again in 2014 (Fig. 4). Spatially, the littoral area of Eastern Lake Taihu was covered with dense aquatic vegetation that extended toward the center of the bay (Fig. 3). The study identified several areas of special interest where major changes have occurred. Generally, aquatic vegetation mapping showed that the deeper, central parts of the bay revealed larger changes compared with the near-shore environment. For example, before 2008, the entire bay area had a VPF above 0.5. However, the center of the bay has had a VPF below 0.5 since 2009, showing a marked decrease in aquatic vegetation cover.

### Seasonal variation in aquatic vegetation

**Figure 5:**
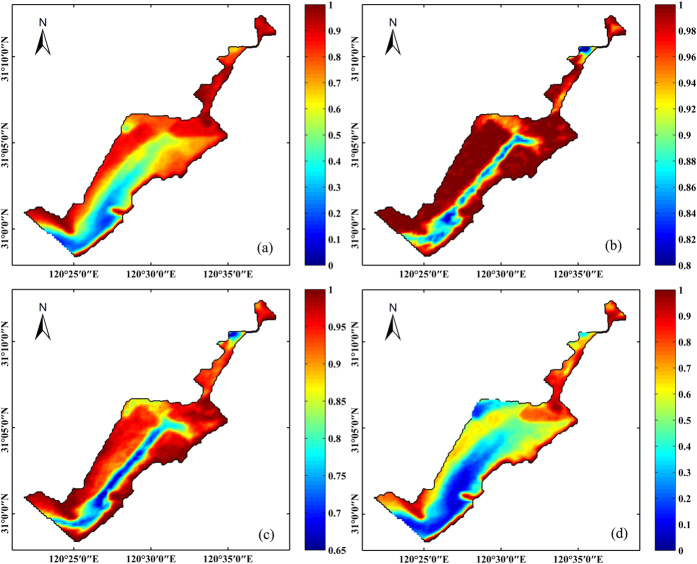
MODIS-Aqua-derived (2003–2014) seasonal VPF in spring (**a**), summer (**b**), autumn (**c**), and winter (**d**).

Figure 5 shows the spatial distribution of VPF of four seasons. The VPF values in spring, summer, autumn and winter were 0.775 ± 0.120, 0.970 ± 0.031, 0.869 ± 0.079, and 0.551 ± 0.129, respectively. Statistically, the VPF presented significant seasonal differences among the four seasons according to the order summer > autumn > spring > winter (Fig. 5). This finding, based on the results of a one-way analysis of variance (ANOVA, *p* < 0.05), partially reflected the life history characteristics of the aquatic vegetation in Eastern Lake Taihu. In winter, most areas of the bay had a VPF below 0.5 (Fig. 5a), which resulted in a substantial amount of sediment resuspension and high turbidity due to low water depth and strong wind waves^6^.

### Correlations between water quality and aquatic vegetation

**Figure 6:**
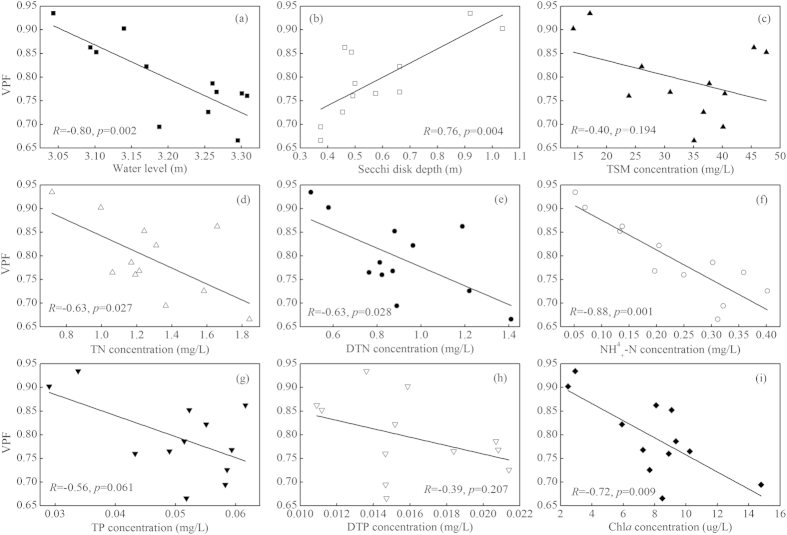
Linear relationships between WL (**a**), SDD (**b**), TSM (**c**), TN (**d**), DTN (**e**), NH_4_^+^-N (**f**), TP (**g**), DTP (**h**), and Chl*a* (**i**) concentrations and the VPF.

The potential factors controlling changes in aquatic vegetation cover in Eastern Lake Taihu were first investigated through a simple linear correlation analysis of the VPF with nine aquatic environmental and water quality variables. Significant negative correlations were observed between the VPF and WL, TN, DTN, NH_4_^+^-N, and Chl*a* concentrations (Fig. 6a,d,e,f,i). In addition, notable but not significant negative correlations were observed between the VPF and the TSM, TP, and DTP concentrations (Fig. 6c,g,h). In contrast, a significant positive correlation was observed between VPF and SDD (Fig. 6b). The significant negative correlation between the VPF and WL in Eastern Lake Taihu in this study differed slightly from the findings of a previous study, which had reported no significant correlations between yearly mean WL and aquatic vegetation area^45^. A possible explanation of this apparent discrepancy was that the limited TM data (a total of 52 remotely sensed images used from 1989 to 2010) could not map the yearly variation of the aquatic vegetation in Lake Taihu^45^.

Because the nine variables are inter-correlated (Table 2), differentiating the relative contributions of the individual variables to the VPF is difficult based solely on the linear correlations between the VPF with nine variables (Fig. 6). We used the definition of dispersion importance to obtain the relative contribution rate of every variable excluding the effects of two auto-correlated variables (Chl*a* and DTN) (Table 3) through the multiple linear regression. The multiple linear regression equation was 0.732SDD/WL + 0.003TSM − 0.050TN − 0.336 NH_4_^+^-N + 0.250TP − 2.139DTP + 0.732 (*R*^2^ = 0.89, *p* = 0.03). The relative contributions of the variables to explaining the total variance were SDD/WL > NH_4_^+^-N > TN > DTP > TP > TSM. Therefore, SDD/WL and NH_4_^+^-N were the two most important variables, explaining 60.12% of the total *R*^2^ of the multiple linear regression.

### Classification and regression trees analysis of VPF

**Figure 7:**
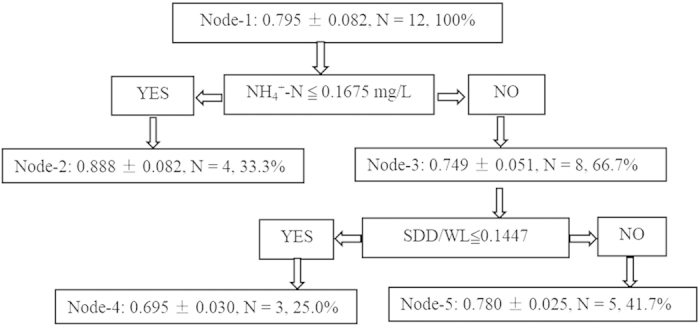
Classification and regression trees for VPF. The groups are represented by the mean, standard deviation, number of samples (years) and percentage (%) of total measurements.

In addition to the analysis of the relative importance based on the linear relationship excluding the effects of auto-correlated variables, the non-linear CART model was used to extract key variables and thresholds in this study. The final CART model showed that a root node (Node-1) was a starting point that included all data (*N* = 12 years, data = 100%) (Fig. 7). NH_4_^+^-N concentration was the most important factor strongly affecting the variability of VPF in Eastern Lake Taihu. The mean VPF for this group was 0.795 ± 0.082. The observations were split (threshold value = 0.1675 mg/L) further based on the high and low NH_4_^+^-N concentration conditions. During the low NH_4_^+^-N concentration condition, the ratio of SDD/WL was another important factor that affected VPF in Lake Taihu. A significant increase in SDD/WL (>0.1447) resulted in the high VPF condition (node = 5, mean = 0.780 ± 0.025, percentage = 41.7%) at this node relative to Node 3. In summary, high NH_4_^+^-N concentration, high SDD/WL and low SDD/WL controlled VPF by approximately 33.3%, 25.0% and 41.7%, respectively, in Eastern Lake Taihu from 2003 to 2014. The CART model showed that NH_4_^+^-N and SDD/WL were the most important factors controlling VPF variability in Eastern Lake Taihu, which was consistent with the results of the relative contributions of the individual variables to the VPF.

## Discussion

### Advantages of aquatic vegetation identification using remote sensing

It is increasingly recognized that efforts to understand ecological resilience and facilitate the management and recovery of aquatic vegetation must ascertain the large-spatial-scale and temporal dynamics rather than considering only simple station data and static cover types^46^. However, the traditional method for investigating aquatic vegetation using an aquascope and rake cannot map variables over large spatial scales and accumulate long-term data on aquatic vegetation dynamics, although field survey methods can provide highly detailed information. Therefore, the potential of using satellite remote sensing technology to detect and assess aquatic vegetation dynamics has been demonstrated in many previous studies including mapping spatial-temporal pattern and biomass^47^,^48^, spectral discrimination of aquatic vegetation species^48^,^49^, and spectral distinctions between aquatic vegetation and other aquatic habitats^24^,^50^. In addition, remote sensing can markedly decrease aquatic vegetation data acquisition costs. The satellite data used for these purposes include satellite multi-spectral moderate spatial resolution data (TM, ETM, MODIS, MERIS), satellite multi-spectral high-spatial resolution data (Quickbird 2), and airborne hyper-spectral (CASI 2) data^22^,^23^,^24^,^25^,^26^,^48^.

Our study used the VPF method based on MODIS data to map the changes in distribution patterns of aquatic vegetation in Eastern Lake Taihu. Frequently repeated satellite image acquisition, in combination with long-term site-specific observations of water quality, has a clear advantage for discussing the temporal and spatial dynamics and potential controlling factors of aquatic vegetation relative to other mapping techniques. However, our study shows only that changes in aquatic vegetation cover have occurred and cannot identify the community succession of aquatic vegetation because a pixel size and mapping unit less than 10–15 m would have been required to accurately map species composition^51^. Further work is needed to identify the community composition change of aquatic vegetation using satellite multi-spectral high-spatial resolution data or airborne hyper-spectral data.

### Controlling factors of aquatic vegetation

Previous studies have demonstrated the link between water environment conditions and aquatic vegetation growth in experimental, observational and remote sensing studies in numerous locations worldwide^45^,^52^,^53^. Although some studies have reported the temporal and spatial dynamics of aquatic vegetation in Lake Taihu^20^,^26^,^45^, the affecting mechanism of environment conditions on aquatic vegetation degradation are far from being fully understood. Our analyses and understanding of aquatic vegetation changes from 1998 to 2014 based on both long-term site-specific observation and remote sensing monitoring data show that aquatic vegetation coverage is likely influenced by a variety of factors. Lake Taihu is a complex ecosystem that has changed measurably over the past thirty years. Rapid population growth and economic development in the lake’s watersheds have resulted in increasing lake eutrophication, phytoplankton biomass and sediment input. All nine aquatic environmental and water quality variables were closely associated with the changes in aquatic vegetation (Fig. 6), as widely observed in other similar studies^14^. However, few studies can clarify the key controlling factors and relative contribution rate of every factor to aquatic vegetation.

The relative importance analysis and CART model conducted in our study indicated that NH_4_^+^-N concentration and SDD/WL were the two most important variables related to VPF; therefore, the changes in the aquatic vegetation of Eastern Lake Taihu were primarily controlled by eutrophication (nutrients and biomass) and hydrologic conditions (WL and underwater light climate). These findings, combined with our linear regression results, imply that the increased trophic level in Eastern Lake Taihu enhanced phytoplankton biomass. Both increased WL and decreased SDD caused by the increase in phytoplankton biomass and TSM resulted in decreases in the ratio of euphotic depth to water depth and further bottom darkening in this area. Healthy and environmentally harmonious values of this ratio are vital to the growth and distribution of aquatic vegetation and even the shift from macrophyte to phytoplankton dominance^39^,^54^. Finally, aquatic vegetation in Eastern Lake Taihu was inhibited and showed a decreasing trend in our study period due to increased eutrophication and a deteriorated underwater light environment.

Compared with phosphorus concentration, nitrogen concentration, particularly ammonia concentration, contributed more to the degradation of aquatic vegetation in Eastern Lake Taihu (Fig. 6, Table 3). Many previous experiment and field studies have shown that high nitrogen concentration, particularly ammonia concentration, caused damage to and loss of aquatic vegetation in shallow lakes^55^,^56^,^57^. Our macro-scale correlation analysis and CART model based on the site-specific observation and remote sensing mapping in Eastern Lake Taihu confirmed this conclusion.

In addition, although we have no long-term observation data of sediment, we did not neglect the potential important roles of lake sediments in driving the nutrient cycle and degradation of SAV in shallow lakes. Sediment is considered an important nutrient source for rooted aquatic vegetation, and fertile sediment plays a positive role in the growth, development, reproduction and germination of aquatic vegetation^2^,^20^,^58^. For example, a previous study showed that depth of soft sediments and nutrient variables were the major factors determining the distribution, growth and community composition of SAV in Lake Taihu^20^.

### Implications for lake ecological restoration

To determine the specific and practically feasible management measures to preserve and restore aquatic vegetation, the quantitative links between the habitat environment and vegetation response must be identified. Our study showed that the habitat environment of aquatic vegetation deteriorated continuously and that the VPF was significantly correlated with intensified eutrophication due to the increase in nitrogen and phosphorus concentrations and deteriorated underwater light environment due to the increased WL, TSM, and Chl*a* concentration and to the decreased SDD in Eastern Lake Taihu. The identification of the controlling factors of aquatic vegetation in Eastern Lake Taihu will help the manager make decisions for recovering aquatic vegetation. In addition, the empirical relationships and CART model developed in this study can be used to assess and forecast the effects of changes in eutrophication and light availability on the status of aquatic vegetation and to help define restoration targets.

Our study showed that nitrogen concentration overrided phosphorus concentration in the degradation of aquatic vegetation in Eastern Lake Taihu, which provided new evidence for nitrogen reduction for aquatic vegetation recovery and eutrophication control. Previous nutrient loading analyses, nutrient addition bioassays and nutrient cycling studies showed that both nitrogen and phosphorus input reductions were required for long-term eutrophication and cyanobacterial bloom control in Lake Taihu^59^,^60^ because eutrophication maybe affect SAV via the increase in the phytoplankton biomass, and further to decrease the SDD and light availability in water bottom^61^. However, although the TP concentration remained lower than the threshold of macrophyte-dominated clear water ecosystem collapse according to the stable state theory, the gradual decrease in the VPF has indicated that Eastern Lake Taihu may currently be close to the critical stage of a stable shift^8^. Many studies have showed that the shift from the macrophyte-dominated clear state to the phytoplankton-dominated turbid stable state can often result in persistent poor water quality in many shallow lakes^8^. However, reducing the nutrient concentrations to the level at which the collapse occurred is often insufficient to restore the macrophyte-dominated clear state. Indeed, the restoration of the macrophyte-dominated clear state occurs at substantially lower nutrient levels than those at which the collapse of the vegetation occurred^7^.

Because the shift from a macrophyte-dominated clear water ecosystem to a phytoplankton-dominated turbid water ecosystem typically lacks massive, marked early-warning signals, special attention should be given to precipitating events rather than to the underlying loss of resilience. Indeed, once eutrophication begins to impact aquatic vegetation, a self-perpetuating macrophyte-dominated clear water ecosystem can be visualized. Many stochastic events that are typically difficult to predict or control can easily trigger state shifts (such as typhoon, high WL, droughts or disease outbreaks)^7^. Therefore, lake management and restoration efforts of Eastern Lake Taihu should seriously consider drastically reducing nutrient input as a key factor to facilitate restoration. Many controlling measurements have been implemented to reduce eutrophication, some monitoring data have showed nutrient load reductions in many inflowing rivers of Lake Taihu, and nutrient concentrations in Eastern Lake Taihu have indicated a decrease from 2010 (Fig. 2) since the implementation of the Major Projects on Control and Rectification of Water Body Pollution in 2008; however, variable nutrient sources and internal cycles have not markedly improved the water quality of Eastern Lake Taihu. Therefore, further exogenous nutrient control and watershed management should be strengthened to facilitate the restoration of Lake Taihu.

In addition, the significant negative correlation between WL and VPF and the positive correlation between SDD and VPF, and SDD/WL as the key controlling factor of VPF indicated that the improvement of underwater light availability was also crucial to the growth, distribution and recovery of aquatic vegetation in many turbid shallow waters^39^,^53^,^62^. In the past twenty years, the increase in the WL and the decrease in the SDD have been accompanied by a continued deterioration of the underwater light environment in Eastern Lake Taihu. Therefore, as an additional measurement, the WL may be lowered, as this lowering can increase the light availability and thereby create a window of opportunity for the establishment of SAV^52^,^63^. Although difficult in practice, we strongly appeal that incidental temporary lowering of the WL should especially be considered in spring in the absence of the flood pressure in Lake Taihu because enabling sufficient light to reach the bottom is a prerequisite for SAV germination in spring.

## Conclusions

This study demonstrates the changes in aquatic vegetation in response to increased eutrophication and deteriorated underwater light climate in Eastern Lake Taihu by combining long-term site-specific observations of water quality and temporal-spatial patterns of aquatic vegetation from remote sensing mapping. Our study indicates that several water quality parameters associated with aquatic vegetation growth have deteriorated from 1998 through 2014 in Eastern Lake Taihu due to eutrophication, which resulted in the degradation of aquatic vegetation. Of all habitat environment factors considered in this study, NH_4_^+^-N concentration and SDD/WL were the most important controlling factors of VPF based on the relative importance partition analysis and CART model.

Although many controlling measurements have been implemented to reduce eutrophication and some monitoring data have showed nutrient load reductions in many inflowing river of Lake Taihu since the implementation of the Major Projects on Control and Rectification of Water Body Pollution in 2008, variable nutrient sources and internal cycles have not markedly improved the lake water quality of Eastern Lake Taihu. Continued population growth, economic development around Lake Taihu and the stressors of climate warming will likely hinder the effectiveness and success of these projects. However, the strong negative correlation between the VPF and nutrient concentrations suggests that further nutrient reductions will be necessary for aquatic vegetation restoration in Eastern Lake Taihu. Examples of areas where water quality has recovered in a holistic manner are needed to facilitate and encourage restoration efforts in Lake Taihu. In addition, lowering the WL to increase SDD/WL and underwater light availability is a potential method of promoting aquatic vegetation recovery in large shallow lakes.

## Additional Information

**How to cite this article**: Zhang, Y.L. *et al*. Aquatic vegetation in response to increased eutrophication and degraded light climate in Eastern Lake Taihu: Implications for lake ecological restoration. *Sci. Rep*. **6**, 23867; doi: 10.1038/srep23867 (2016).

## Figures and Tables

**Table 1:** Descriptive statistics for nine aquatic environmental and water quality variables (WL, SDD, TSM, TN, DTN, NH_4_^+^-N, TP, DTP, and Chl*a*) and the VPF.

	WL m	SDD m	TSM mg/L	TN mg/L	DTN mg/L	NH_4_^+^-N mg/L	TP mg/L	DTP mg/L	Chl*a μ*g/L	VPF
Min	3.02	0.38	11.89	0.47	0.29	0.034	0.029	0.011	2.18	0.666
Max	3.31	1.10	47.62	1.84	1.41	0.402	0.062	0.021	14.79	0.935
Mean	3.19	0.68	27.49	1.11	0.83	0.179	0.049	0.016	6.52	0.795
Standard deviation	0.09	0.24	12.51	0.38	0.31	0.124	0.010	0.004	3.54	0.082

**Table 2:** Coefficient of determination and significance level of Pearson correlations for nine aquatic environmental and water quality variables.

	WL	SDD	TSM	TN	DTN	NH_4_^+^-N	TP	DTP	Chl*a*
WL	1.00								
SDD	0.21^NS^	1.00							
TSM	0.02^NS^	0.64**	1.00						
TN	0.09^NS^	0.55**	0.31^NS^	1.00					
DTN	0.11^NS^	0.55**	0.31^NS^	0.96**	1.00				
NH_4_^+^-N	0.63**	0.49*	0.21^NS^	0.23^NS^	0.26^NS^	1.00			
TP	0.06^NS^	0.61**	0.60**	0.48*	0.50^NS^	0.28^NS^	1.00		
DTP	0.41*	0.00^NS^	0.01^NS^	0.01^NS^	0.00^NS^	0.31^NS^	0.02^NS^	1.00	
Chl*a*	0.18^NS^	0.71**	0.54**	0.16^NS^	0.12^NS^	0.46*	0.40**	0.00^NS^	1.00

NS: not significant; ^*^p < 0.05; ^**^p < 0.01.

**Table 3:** Relative contribution rate of six variables to the VPF.

	SDD/WL	NH_4_^+^-N	TN	DTP	TP	TSM
Contribution rate (%)	30.12	30.00	14.18	11.66	8.24	5.80
